# FAM20C: A key protein kinase in multiple diseases

**DOI:** 10.1016/j.gendis.2023.101179

**Published:** 2023-11-23

**Authors:** Rui Zhang, Yanming Ren, Yan Ju, Yuekang Zhang, Yan Zhang, Yuan Wang

**Affiliations:** a Department of Neurosurgery, State Key Laboratory of Biotherapy and Cancer Center, West China Hospital, Sichuan University, Chengdu, Sichuan 610041, China; b National Clinical Research Center for Geriatrics, West China Hospital, Sichuan University, Chengdu, Sichuan 610041, China

**Keywords:** Biological functions, Biomineralization, Cancer progression, FAM20C, Protein phosphorylation

## Abstract

Family with sequence similarity 20 C (FAM20C) is a Golgi protein kinase that phosphorylates the serine residue in the S-x-E/pS motif of target proteins. FAM20C phosphorylates most secreted proteins, which play important roles in multiple biological processes, including cancer progression, biomineralization, and lipid homeostasis. Numerous studies have documented the potential contribution of FAM20C to the growth, invasion, and metastasis of glioma, breast cancer, and other cancers, as well as to the mineralization process of teeth and bone. In addition, FAM20C has been found to be associated with the occurrence and development of certain cardiovascular diseases and endocrine metabolism disorders. It raises hopes that understanding the disease-specific mechanisms of FAM20C may hold the key to developing new strategies for these diseases. This review comprehensively covers the existing literature to provide a summary of the structure and biological functions of FAM20C, with a particular focus on its roles in the disease context.

## Introduction

Phosphorylation is a common posttranslational modification mediated by kinases.^1^ Protein phosphorylation refers to transferring a phosphate group from adenosine triphosphate or guanosine triphosphate to the specific amino acid residue of target proteins through the catalytic action of protein kinases, thereby regulating its biological activities.^2^ Protein phosphorylation is crucial in various physiological processes such as cellular signal transduction, tissue growth, and development. Dysregulation of this process can result in related diseases, including cancers, diabetes, and Alzheimer's disease.^2^^,^^3^ While most protein kinases target intracellular proteins, only a few have been found to phosphorylate secreted proteins.

Secreted proteins play significant roles in various pathophysiological processes, including extracellular matrix remodeling, wound repair, and disease development.^4^^,^^5^ The studies of secreted protein phosphorylation date back to the late 19th century, when it was discovered that casein could be phosphorylated.^6^ Casein kinases represent three distinct types of kinases: casein kinase 1, casein kinase 2, and Golgi-casein kinase/FAM20C.^7^ FAM20C, first identified in 1972, is an important kinase involved in the phosphorylation of several proteins in the secretory pathway, contributing to multiple pathophysiological processes.^8^^,^^9^ In fact, phosphoproteomic studies have demonstrated that FAM20C is responsible for the majority of phosphoproteins.^5^

FAM20 family is an evolutionarily conserved protein family with three members in mammals, namely FAM20A, FAM20B, and FAM20C.^8^ However, FAM20A is typically not expressed in invertebrates.^10^ For example, *Caenorhabditis elegans* possesses only one member, which is the FAM20C orthologue.^10^ In contrast, zebrafish and pufferfish have five and six family members, respectively, primarily due to an increase in the FAM20C subfamily members.^8^ FAM20C is generally expressed in tissues such as the brain, breast, pancreas, teeth, and bone, and exerts significant functions in these tissues.^5^ Therefore, this review provides an overview of the structure and biological roles of FAM20C, with a particular emphasis on its association with multiple cancers. Additionally, we also discuss potential substrates that may contribute to cancer progression.

## The structure of FAM20C

**Figure 1:**
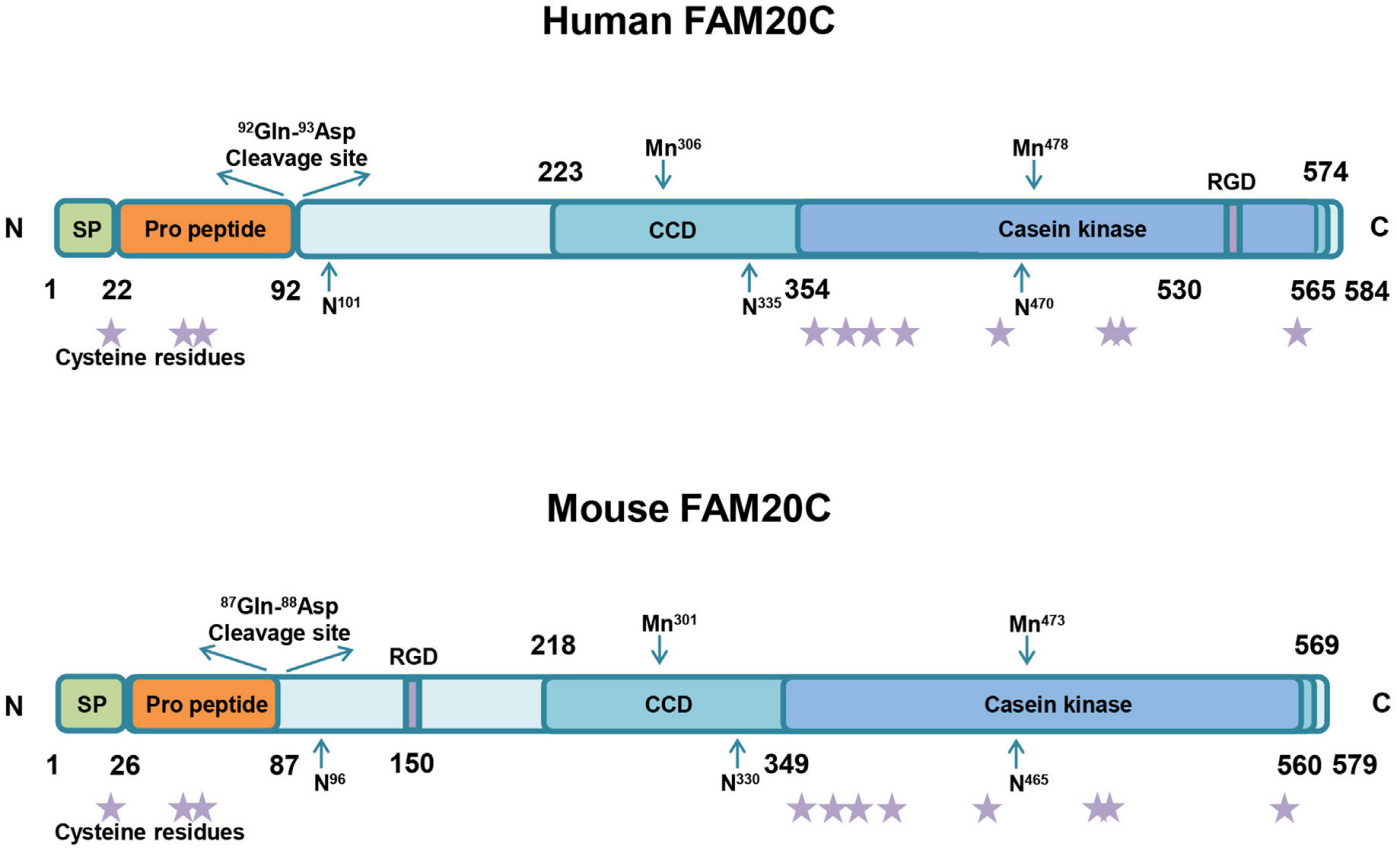
The structures of human and mouse FAM20C. Human FAM20C consists of 584 amino acids (aa), with an N-terminal signal peptide (SP) composed of 22 aa, and a conserved C-terminal domain (CCD) composed of 350 aa. CCD contains a casein kinase (CK) domain ranging from residue 354 to 565. Mouse FAM20C consists of 579 aa, with an SP comprised of 26 aa and a CCD comprised of 350 aa. CCD also contains a CK domain ranging from residue 349 to 560. Furthermore, the structure of FAM20C contains a cleavage site, an integrin-bound tripeptide (arginine-glycine-aspartate, RGD), two Mn2^+^ binding sites, three N-glycosylation sites, and 11 cysteine residues. The stars represent eight cysteine residues that are conserved within different species.

FAM20C protein sequences are highly similar among different species. For instance, there is 85% identity and 91% similarity between human and mouse FAM20C sequences.^11^ The human *FAM20C* gene that spans 72410bp is located on chromosome 7p22.3.^12^ Ten exons are included in the *FAM20C* gene, and only exon one shows size differences between humans and mice.^8^ The mRNA length of FAM20C is 3176bp, and it contains 1755 nucleotide coding sequences.^8^^,^^12^ FAM20C consists of 584 amino acids.^13^^,^^14^ The N-terminus of FAM20C has a signal peptide composed of 22 amino acids, which facilitates FAM20C targeting the secretory pathway.^14^ The C-terminus harbors a conserved C-terminal domain composed of 350 amino acids.^8^^,^^14^, ^15^, ^16^ The conserved C-terminal domain includes a casein kinase domain ranging from residue 354 to 565, spanning 222 amino acids.^8^^,^^12^ The N-lobe and C-lobe structures that are the features of protein kinases are included in the kinase core.^13^ Mouse FAM20C, also known as dentin matrix protein 4, consists of 579 amino acids, with a 26-amino acid N-terminal signal peptide and a 350-amino acid conserved C-terminal domain.^15^^,^^17^ The conserved C-terminal domain also includes a casein kinase domain ranging from residue 349–560.^8^ The structure of FAM20C also contains 11 cysteine residues, an integrin-bound tripeptide (arginine-glycine-aspartate), and three different N-glycosylation sites.^17^, ^18^, ^19^ Eight cysteine residues in the conserved C-terminal domain are completely conserved within different species and may contribute to the formation of intermolecular or intramolecular disulfide bonds.^8^ Arginine-glycine-aspartate is crucial for mediating cellular adhesion and migration through interacting with integrin.^20^ The three asparagine residues that can be N-glycosylated play important roles in FAM20C folding and secretion.^5^^,^^18^ Furthermore, the residues within the Mn^2+^ and adenosine triphosphate binding sites are critical for nucleotide binding and catalysis (Fig. 1).^7^^,^^21^ Interestingly, the crystal structure of the FAM20C orthologue in *Caenorhabditis elegans* displays an atypical kinase fold surrounded by a shell-like structure formed by a novel insertion domain and an N-terminal segment.^21^ The base of the C-lobe is formed by the N-terminal segment, which encircles the lower half of the molecule, and the N-lobe is covered by a cap-like structure that is formed by the insertion domain.^21^ Because this domain is concealed in the N-lobe, FAM20C often fails to be identified as a protein kinase by sequence analyses.^21^ Further investigations into the structure of FAM20C can offer valuable insights into its biological functions and facilitate the identification of more effective therapeutic targets for related diseases.

## The biological functions of FAM20C

Although FAM20A, FAM20B, and FAM20C belong to the same family, their biological functions are completely different.^22^ FAM20A is a pseudokinase lacking kinase activity, while FAM20B is a glycan kinase that phosphorylates xylose residues within proteoglycans, a process essential for proteoglycan biosynthesis.^22^ FAM20C functions as a protein kinase and is known to phosphorylate more than 100 substrates. Specifically, FAM20C targets the serine (Ser) residue of S-x-E/pS motif in its target proteins that play essential roles in cancer progression, biomineralization, endoplasmic reticulum (ER) homeostasis, and other processes.^5^^,^^23^ FAM20C also can recognize the S-x-Q-x-x-D-E-E motif and R-G-D-S-V-V-Y-G-L-R motif, suggesting that its substrates may be more extensive, and it may be involved in more complex biological processes.^20^^,^^24^ Additionally, FAM20C can phosphorylate other amino acid residues such as the specific threonine residue of the neuroendocrine chaperone 7B2.^25^

The activity of FAM20C is dynamically regulated by FAM20A. FAM20C and FAM20A can form a heterodimeric complex that significantly promotes the activity of FAM20C to phosphorylate-related substrates.^19^^,^^26^ Two heterodimers can further form a hetero-tetrameric complex, but it is still unclear which type actually exists *in vivo*.^6^ FAM20A exhibits a greater affinity for FAM20C and allosterically activates FAM20C more effectively than FAM20C itself.^19^^,^^26^^,^^27^ FAM20A has some special sites, including Ile214A, Ile255A, Phe251A, Phe252A, and Leu365A, which enable it to interact with FAM20C effectively.^19^^,^^22^ Cofactors also affect FAM20C's activity. FAM20C exhibits higher selectivity for Mn^2+^ and Co^2+^ than Mg^2+^, but it still utilizes Mg^2+^ in some specific physiological processes.^6^^,^^28^ The ability of FAM20C to use Mg^2+^ as a cofactor is considerably increased by sphingosine and sphin-gosine-1-phosphate.^29^^,^^30^ The addition of sphingosine increases the activity of FAM20C *in vitro* by eight times, resulting in a three-fold rise in Vmax and a corresponding three-fold fall in Km, which suggests sphingosine acts specifically as a FAM20C activator.^29^^,^^30^ Site-1 protease, a crucial regulator of cholesterol homeostasis, governs the secretion and activity of FAM20C.^31^ Specifically, site-1 protease can modulate the proteolysis of mature FAM20C and then promote its activity.^31^ FAM20C devoid of site-1 protease can impede its secretion and functional properties.^32^ In addition, fingolimod, an immunosuppressant drug for multiple sclerosis, can stimulate the activity of FAM20C.^30^ Given that fingolimod and sphingosine share a structural similarity, this may not be surprising.^30^ Interestingly, the majority of kinase inhibitors tested so far on FAM20C, including staurosporine, fail to inhibit FAM20C.^30^

## The role of FAM20C in multiple cancers

**Figure 2:**
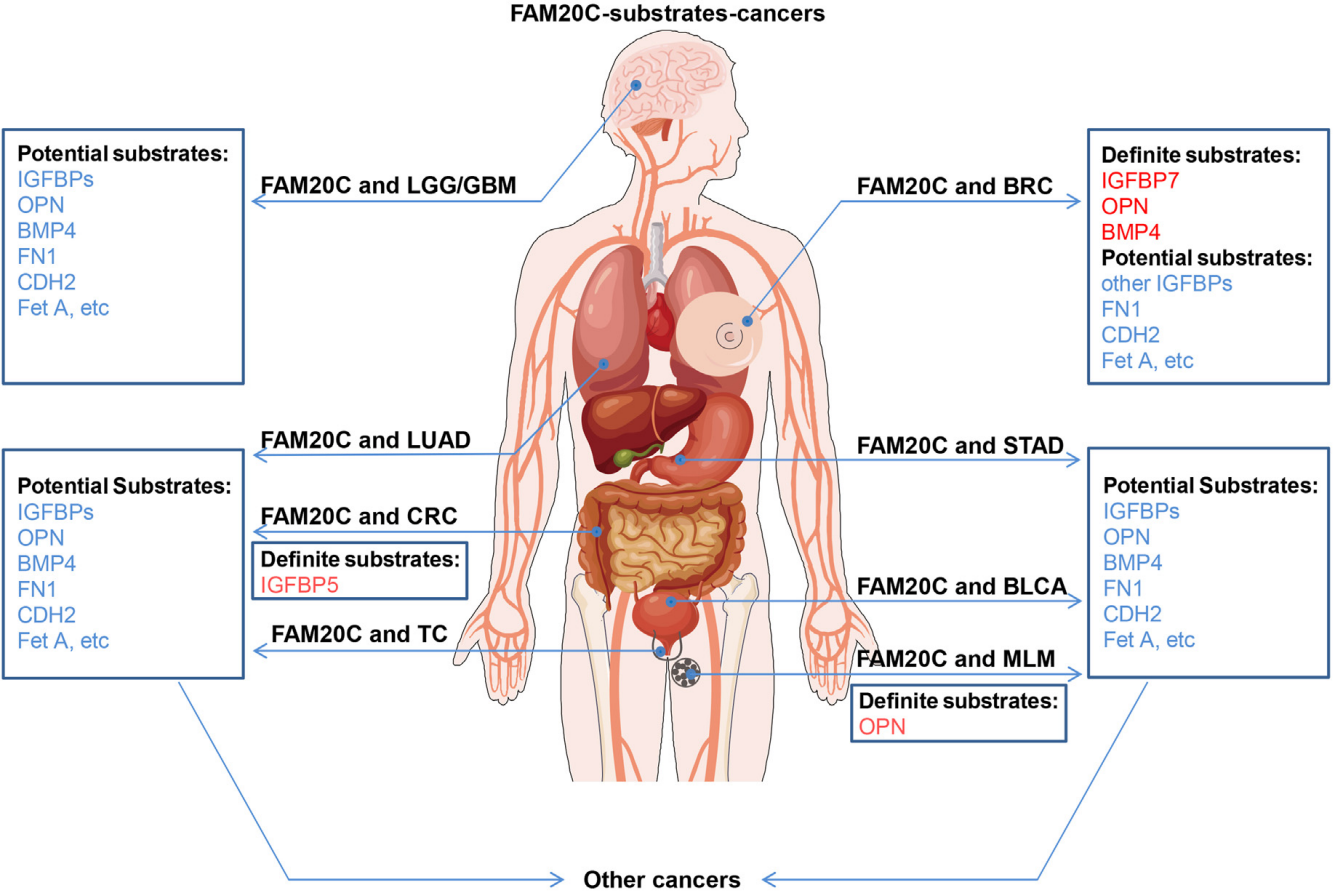
FAM20C and its substrates in multiple cancers. FAM20C has been identified to be directly or indirectly associated with eight types of cancers. If it has been reported in the literature that FAM20C can interact with a substrate to promote cancer progression, this substrate is the definite substrate. If there is no literature reporting that FAM20C can interact with a substrate to promote cancer progression, this substrate is the potential substrate. The definite substrates are marked in red and the potential substrates in blue.

**Figure 3:**
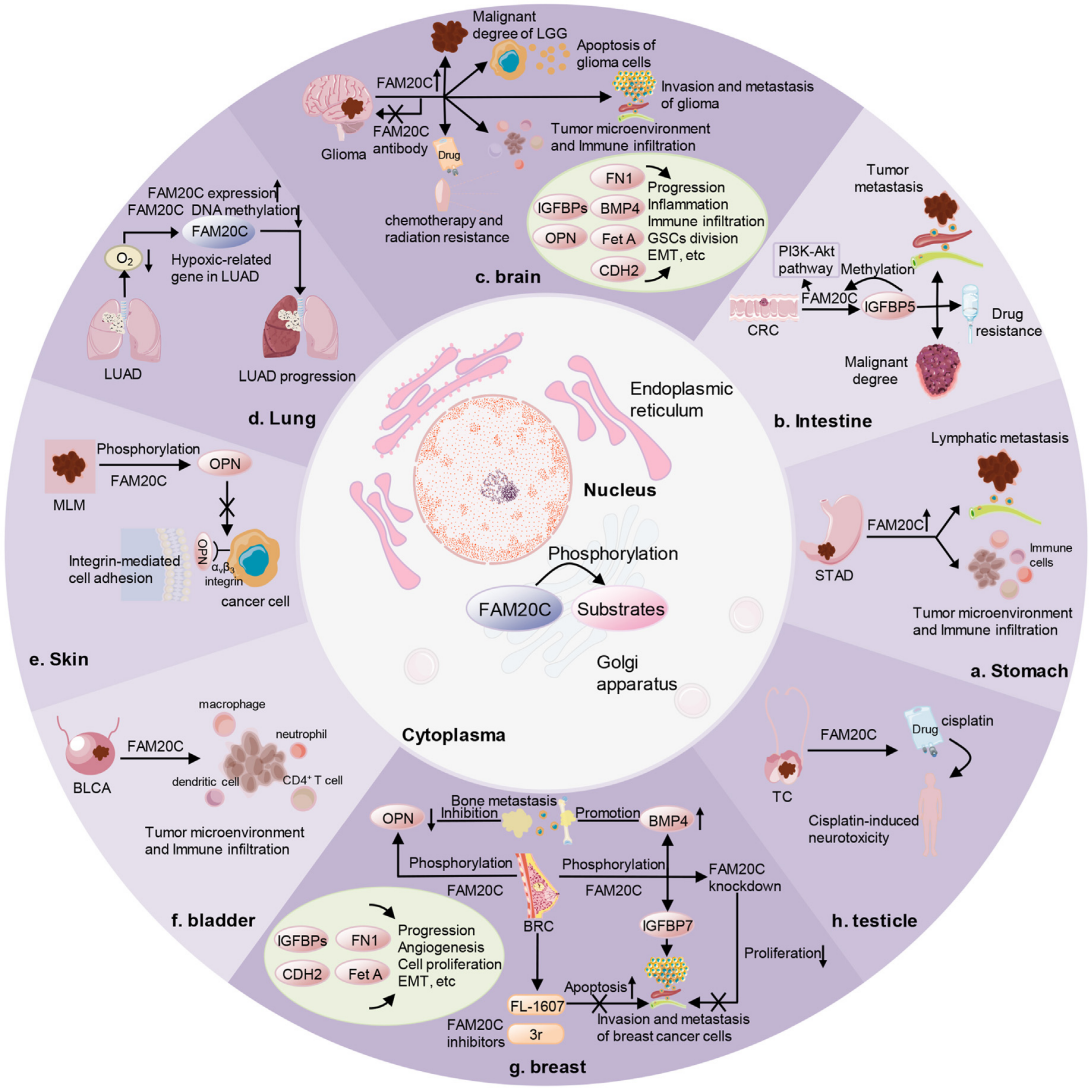
The roles of FAM20C in multiple cancers. FAM20C participates in multiple biological processes of eight types of cancers. It has been extensively studied in glioma and breast cancer (BRC), where its potential substrates have also been investigated in great detail.

The invasion and metastasis of tumor cells are very complicated processes. Protein kinases have been identified to play key roles in cancer progression, and their inhibitors are also expected to be used in the treatment of related cancers.^7^ Database analyses have revealed that FAM20C expression is increased in multiple malignant tumors, including central nervous system tumors, breast cancer (BRC), lung adenocarcinoma, pancreatic tumors, and lymphoma.^33^ Moreover, several FAM20C substrates such as insulin-like growth factor-binding proteins (IGFBPs), osteopontin (OPN), bone morphogenetic protein 4, fibronectin 1, and fetuin-A (Fet A) are associated with the apoptosis, invasion, and metastasis of tumor cells.^5^^,^^22^^,^^34^, ^35^, ^36^ The IGFBP family includes seven members that play roles in tumor development, metabolism, and other processes.^37^ OPN belongs to the small integrin binding ligand-N-linked glycoprotein (SIBLING) family and is involved in many pathophysiological processes such as cell signal transduction, biomineralization, and tumor development.^38^ As a member of the transforming growth factor β family, bone morphogenetic protein 4 can affect cell proliferation and differentiation of multiple organs, and tumor development.^35^^,^^39^ Fibronectin 1, a member of the fibronectin family, participates in various biological processes, including cell adhesion and migration, and cytoskeletal organization.^40^ Fetuin belongs to the cystatin superfamily and includes Fet A and Fet B.^41^ Fet A is a glycoprotein that is synthesized primarily by the liver in adults.^42^ Phosphorylated Fet A can affect glucose metabolism and cancer progression.^43^^,^^44^ These substrates mentioned above have been widely demonstrated to be related to the invasion and metastasis of multiple cancers such as glioma, BRC, lung cancer, and colorectal cancer (CRC) (Figure 2, Figure 3).^45^, ^46^, ^47^, ^48^, ^49^, ^50^, ^51^, ^52^, ^53^, ^54^

The mechanisms that FAM20C contributes to cancer progression have been widely reported. FAM20C promotes the invasion and metastasis of multiple cancers by phosphorylating related substrates.^5^ In addition, FAM20C is associated with the infiltration levels of CD4^+^ T cells, macrophages, neutrophils, and dendritic cells in the tumor microenvironment of bladder urothelial carcinoma, lower-grade glioma, and stomach adenocarcinoma, indicating that FAM20C may affect the degree of immune infiltration and the activation of different immune cells, thus influencing cancer progression through tumor microenvironment.^33^ Specific mechanisms may include regulating the polarization of tumor-associated macrophages, activating regulatory T cells, inducing T cell depletion, and regulating T helper cells.^33^ It is speculated that FAM20C can affect more cancer progression through the tumor microenvironment. Epithelial-mesenchymal transition is a cellular process in which cells gain mesenchymal traits while losing their epithelial features.^55^ It is related to the occurrence, invasion, metastasis, and resistance to therapy of multiple cancers, including glioma and BRC.^33^^,^^55^, ^56^, ^57^, ^58^, ^59^ Cadherin-2 is a phosphorylated substrate of FAM20C and a marker of epithelial-mesenchymal transition, so FAM20C may be involved in epithelial-mesenchymal transition through cadherin-2, thereby influencing related cancers (Figure 2, Figure 3).^5^^,^^33^^,^^55^

Numerous studies have demonstrated the involvement of FAM20C in cancer progression, highlighting its potential as a promising therapeutic target for the prevention and treatment of related cancers.^23^

## FAM20C and glioma

Glioma is the most common malignant tumor of the central nervous system and can be classified into lower-grade glioma (WHO grade I and WHO grade II) and higher-grade glioma (WHO grade III and WHO grade IV).^60^ Although extensive research has been conducted in this field, the mechanisms underlying glioma invasion and metastasis remain unclear, and effective treatments are currently limited. The conventional treatment for glioma includes surgical removal of the tumor within the maximum safe range, supplemented by chemotherapy and radiotherapy, but the prognosis remains poor.^61^ Protein kinases have been demonstrated to play important roles in glioma progression.^62^ FAM20C is highly expressed in lower-grade glioma, and its expression is positively correlated with the malignant degree and progression of glioma, while negatively correlated with the prognosis of patients.^33^^,^^61^ Gene set enrichment analysis has revealed that the mechanisms of glioma progression mediated by FAM20C include cell apoptosis, invasion, and metastasis.^61^ A recent study has confirmed that the expression of FAM20C is up-regulated in glioma by long-read RNA sequencing.^63^ FAM20C can promote the invasion of glioma *in vitro*, and FAM20C antibodies can significantly reduce glioma size in animal experiments.^63^ In addition, genome-wide association studies have identified that the variant near the *FAM20C* gene, rs111976262, is associated with adult diffuse glioma that has isocitrate dehydrogenase (*IDH*) mutation, and 1p/19q codeletion, or has *IDH* mutation, 1p/19q codeletion, and telomerase reverse transcriptase (*TERT*) mutation (Table 1 and Fig. 3).^64^^,^^65^

**Table 1:** Literature on FAM20C and glioma.

Authors, published year	Main research methods	Research conclusions	Reference
Eckel-Passow et al., 2020	GWAS analysis, etc.	1. A variant near *FAM20C* gene (rs111976262) is associated with adult diffuse glioma that has *TRET* mutation, *IDH* mutation and 1p/19q codeletion.	64
Du et al., 2020	Cell culture, LASSSO regression analysis, Immunohistochemistry, Functional analysis, Database analysis, etc	1. The expression of FAM20C is positively associated with GBM progression and negatively with prognosis of GBM patients. 2. FAM20C is associated with the disrupted immune response in GBM microenvironment. 3. FN1 may be the substrate of FAM20C.	69
Trendowski et al., 2020	Genotyping and genome-wide analysis, Evaluation of cisplatin sensitivity in silico, etc.	1. Reduced expression of FAM20C can enhance the sensitivity of CNS tumors to cisplatin.	76
Liu et al., 2021	Database analysis, Statistical analysis, etc.	1. The high expression of FAM20C is associated with poor prognosis of LGG patients. 2. The expression of FAM20C is positively associated with immune infiltration level in LGG. 3. CDH2 (a substrate of FAM20C) is one of the markers of EMT that is associated with invasion and metastasis of cancer cells.	33
Feng et al., 2021	Database analysis, Functional enrichment analysis, Gene set enrichment analysis, Immunohistochemistry, etc.	1. The expression of FAM20C is positively associated with LGG progression and negatively with prognosis of LGG patients. 2. The mechanisms of LGG progression mediated by FAM20C includes extracellular matrix receptor interactions, cell apotosis and cell adhesion, etc.	61
Eckel-Passow et al., 2022	GWAS analysis, etc.	1. A variant near *FAM20C* gene (rs111976262) is associated with adult diffuse glioma that has *IDH* mutation and 1p/19q codeletion.	65
Gong et al., 2023	ONT long-read Nanopore RNA sequencing, ATAC-Seq, Transwell invasion assay, Tumor xenografts in nude mice, etc.	1. The expression of FAM20C is upregulated in glioma. 2. FAM20C can promote the invasion of glioma *in vitro*. 3. FAM20C antibody can significantly reduce glioma size *in vivo*. 4. FAM20C is a promising prognostic marker for glioma.	63
Ren et al., 2023	Spatial transcriptomics, Gene ontology enrichment analysis, Orthotopic xenograft, Transwell migration assay, etc.	1. The high expression of FAM20C in RG-like cells mediates the invasive growth of H3K27M-DMG in the neuron-rich invasive region.	75

GBM, glioblastoma; LGG, low-grade glioma; GWAS, genome-wide association study; FN1, Fibronectin 1; CDH2, Cadherin 2; EMT, Epithelial–mesenchymal transition; CNS, central nervous system; LASSO, least absolute shrinkage and selection operator; ATAC-Seq, the Assay for Transposase-Accessible Chromatin with high-throughput sequencing; TRET, telomerase reverse transcriptase; IDH, isocitrate dehydrogenase; RG-like, radial glial stem-like; H3K27M-DMG, diffuse midline glioma-H3K27M mutant.

Glioblastoma (GBM) is the most lethal malignant brain tumor that primarily occurs in adults.^66^ Patients with GBM have an extremely poor prognosis, with a median survival of only 15 months.^67^^,^^68^ *In vitro*, it has been demonstrated that high expression of FAM20C is associated with GBM progression and often indicates a poor prognosis for patients.^69^ FAM20C knockout impairs the ability of GBM-LN229 cells to invade and metastasize (Fig. 2).^69^ It is suggested that FAM20C may promote GBM progression by phosphorylating related substrates. Moreover, the quantity of monocytes and macrophages is greater in the tumor microenvironment of GBM with higher FAM20C expression (Table 1).^69^ Monocytes in the tumor microenvironment can differentiate into tumor-associated macrophages that can promote tumor development, and an increase in the number of tumor-associated macrophages often indicates a poor prognosis.^70^^,^^71^ Therefore, it is indicated that FAM20C may promote GBM progression by affecting the tumor microenvironment (Fig. 3).

Diffuse midline glioma harboring H3K27M mutant is the deadliest malignant brain tumor that primarily occurs in children.^72^ It typically arises in midline brain regions such as the brainstem, cerebellum, and thalamus.^73^ The hallmark of the disease is the mutation, the lysine 27-to-methionine mutation in histone H3 (*H3K27M*), which is frequently combined with platelet-derived growth factor receptor alpha (*PDGFRA*) amplification and tumor protein P53 (*TP53*) mutation.^74^ A recent study has identified a group of radial glial stem-like (RG-like) cells in the neuron-rich invasive region through spatial transcriptomics and single-cell transcriptomic datasets.^75^ In vivo and *in vitro*, it is demonstrated that high expression of FAM20C in RG-like cells mediates the invasive growth of diffuse midline glioma harboring H3K27M mutant in the neuron-rich invasive region.^75^ This study is the first to demonstrate the roles of FAM20C in glioma invasion *in vivo* (Table 1 and Fig. 3). In conclusion, FAM20C can affect glioma progression through many ways and is a promising prognostic marker for glioma. However, its downstream mechanisms remain unclear and deserve further study.

Patients with higher FAM20C levels are more resistant to chemotherapy and radiation.^33^ Meanwhile, a clinical and genome-wide analysis has also revealed that reduced expression of FAM20C can enhance the sensitivity of central nervous system tumors to cisplatin (Table 1 and Fig. 3).^76^ These findings suggest that the expression of FAM20C in central nervous system tumors may influence the sensitivity of tumors to chemotherapy drugs. Therefore, evaluating the relationship between the sensitivity of glioma to temozolomide and other chemotherapy drugs and the levels of FAM20C could provide valuable insights into glioma treatment strategies. The above studies have demonstrated the roles of FAM20C in glioma progression, but further research is worthy of being conducted to identify the substrates and specific mechanisms involved, which could contribute to designing relevant targeted drugs to prevent glioma progression.

## FAM20C and BRC

BRC is one of the most common malignant tumors among women, posing a serious threat to women's health.^47^ Despite the application of various therapeutic methods, the prognosis of BRC remains unsatisfactory.^47^ The underlying etiology and pathogenesis are still unknown. Therefore, it is imperative to study the invasive and metastatic mechanisms of BRC, which will contribute to the treatment of BRC. The association between FAM20C and triple-negative BRC was first reported in 2015.^5^ Cell experiments have demonstrated that FAM20C promotes the migration of BRC MDA-MB-231 cells by phosphorylating IGFBP7, and its knockout attenuates the ability of BRC cells to migrate.^5^ Furthermore, FAM20C knockdown by small interfering RNA can inhibit the metastasis of MDA-MB-231 cells, while mildly affecting proliferation.^77^ Animal experiments have revealed that myeloid FAM20C can phosphorylate OPN and reduce its secretion, thereby inhibiting osteoclast differentiation and bone metastasis of BRC.^35^ However, cell experiments have found that FAM20C in BRC cells can induce osteoclast differentiation and promote bone metastasis of BRC MDA-BoM-1833 cells by phosphorylating bone morphogenetic protein 4 and promoting its secretion (Table 2).^35^ The different effects of FAM20C on myeloid cells and BRC cells, and the distinct changes of OPN and bone morphogenetic protein 4, suggest that the development of related drugs targeting them has great potential to inhibit bone metastasis of BRC. Taken together, these studies have identified the important roles of FAM20C in the invasion and metastasis of BRC (Figure 2, Figure 3).

**Table 2:** Literature on FAM20C and breast cancer.

Authors, Published year	Main research methods	Research conclusions	Reference
Tagliabracci et al., 2015	Detachment assay, Scratch wound-healing assay, Trans -well chemotaxis migration assay, Matrigel invasion assays, etc.	1. FAM20C promotes the migration of BRC MDA-MB-231 cells by phosphorylating IGFBP7. 2. FAM20C knockout weakens the ability of migration of BRC MDA-MB-231 cells.	5
Qin et al., 2016	Network construction, Homology modeling, Cell culture, Molecular dynamics simulations, Cell proliferative assay, Scratch wound-healing assay, etc.	1. A small molecule inhibitor of FAM20C, FL-1607, can inhibit the proliferation of TNBC cells. 2. FL-1607 can inhibit migration and promote the apoptosis of BRC MDA-MB-468 cells	78
Liu et al., 2021	Database analysis, Statistical analysis, etc.	1. FAM20 is highly expressed in BRC. 2. CDH2 (a substrate of FAM20C) is one of the markers of EMT that is associated with invasion and metastasis of cancer cells.	33
Zhao et al., 2021	Combining silicon high-throughput screening with chemical synthesis methods, Cell culture, Transwell migration assay, siRNA transfection, Xenograft tumor model, etc.	1. A small molecule FAM20C inhibitor 3r can promote the apoptosis of BRC MDA-MB-231 cells and inhibit the migration of MDA-MB-231 cells. 2. 3r can inhibit tumor growth of TNBC cells *in vivo*. 3. FAM20C knockdown by siRNA can inhibit the metastasis of MDA-MB-231 cells and mildly affect proliferation	77
Zuo et al., 2021	Mice model, Cell culture, Bone metastasis analyses, Mass spectrometry analyses of phosphopeptides, Cell proliferation and migration assay, Analyses of primary tumor growth, etc.	1. Myeloid FAM20C can phosphorylate OPN and reduce its secretion, thereby inhibiting osteoclast differentiation and bone metastasis of BRC. 2. FAM20C in BRC cells can induce osteoclast differentiation and promote bone metastasis of human BRC MDA-BoM-1833 cells by phosphorylating BMP4 and promoting BMP4 secretion.	35

BRC, breast cancer; TNBC, triple negative breast cancer; siRNA, small interfering RNA; OPN, osteopontin; BMP4, bone morphogenetic protein 4; CDH2, Cadherin 2.

Aiming at the roles of FAM20C in the invasion and metastasis of BRC, some studies have discovered some potential anti-tumor agents targeting FAM20C. Through structural comparison in the compound library, a small molecule inhibitor of FAM20C, FL-1607, has been found to inhibit the proliferation of triple-negative BRC cells.^78^ Meanwhile, it can inhibit the migration and promote the apoptosis of MDA-MB-468 cells.^78^ In addition, a small molecule inhibitor of FAM20C, 3r, is discovered by combining silicon high-throughput screening with chemical synthesis methods.^77^ It can inhibit the migration and promote the apoptosis of MDA-MB-231 cells via the mitochondrial pathway, and inhibit the growth of triple-negative BRC *in vivo* (Table 2 and Fig. 3).^77^ These promising findings strongly support the efficacy of FAM20C inhibitors in the treatment of BRC.

## FAM20C and other cancers

FAM20C has also been implicated in the progression of other cancers. Hypoxia and DNA hypomethylation can affect cancer progression and often predict a poor prognosis.^79^ FAM20C is related to hypoxia in lung adenocarcinoma progression. Hypoxia in lung adenocarcinoma cells can promote the expression of FAM20C and inhibit the DNA methylation of the *FAM20C* gene, thus facilitating lung adenocarcinoma progression.^79^ The ability of OPN to participate in integrin-mediated cell adhesion depends on its phosphorylation state. Human melanoma MDA-MB-435 cells weakly adhere to highly phosphorylated OPN, but strongly adhere to low-phosphorylated OPN.^80^ A cell experiment has revealed that FAM20C can phosphorylate the Ser residue of the R-G-D-S-V-V-Y-G-L-R motif of OPN, which can reduce the interaction between phosphorylated OPN and α_v_β_3_ integrin in MDA-MB-435 cells, thus affecting integrin-mediated cell adhesion.^20^ FAM20C also plays important roles in CRC metastasis. The expression of IGFBP5 is increased in CRC, which can promote lymph node metastasis.^37^ The levels of IGFBP5 are positively correlated with the malignant degree, metastasis, and drug resistance of CRC, and negatively correlated with the prognosis of patients.^37^ Furthermore, IGFBP5 may affect the methylation of insulin-like growth factor 1/2, FAM20C, and other molecules, thereby regulating the activation of the PI3K-Akt signaling pathway and facilitating CRC progression.^37^ It is indicated that FAM20C may participate in the development of CRC through interacting with IGFBP5. Elevated levels of FAM20C are indicative of a poor prognosis for patients with bladder urothelial carcinoma or stomach adenocarcinoma.^33^ Particularly, FAM20C can significantly promote the lymph node metastasis of stomach adenocarcinoma.^33^ In addition, clinical and genome-wide analyses have revealed that FAM20C is associated with the neurotoxicity of cisplatin in testicular cancer (Table 3 and Figure 2, Figure 3).^76^

**Table 3:** Literature on FAM20C and other cancers.

cancers	Authors, published year	Main research methods	Research conclusions	Reference
CRC	Deng et al., 2022	Immunohistochemical analysis, Survival analysis, Database analysis, Methylation-related analysis, Gene set enrichment analysis, etc.	1. The expression of IGFBP5 is associated with the tumor differentiation, tumor metastasis, drug resistance and prognosis of CRC patients. 2. IGFBP5 may affect the methylation of IGF1, IGF2, FAM20C and other molecules, thereby regulating the activation of PI3K-Akt signaling pathway and promoting CRC development.	37
LUAD	Li et al., 2020	Database analysis, GSVA algorithm, Survival analysis, GO analysis, KEGG analysis, PPI analysis, Cell culture and cell treatment, etc.	1. FAM20C has been identified as the hypoxia-related key gene in LUAD progression. 2. Hypoxia in LUAD cells can promote the expression of FAM20C and inhibit the DNA methylation of FAM20C gene, further leading to LUAD progression.	79
MLM	Schytte et al., 2020	Plasmid construction, Protein expression and purification, Kinase assay, Cell adhesion studies, etc.	1. FAM20C can phosphorylate the Ser residue of RGDSVVYGLR of OPN and reduce the interaction with α_v_β_3_ integrin in MDA-MB-435 cells, thus affecting integrin-mediated cell adhesion.	80
TC	Trendowski et al., 2020	Establishment of the multiple severe cisplatin-induced neurotoxicities Phenotype, Phenotype association analysis, etc.	1. FAM20C is associated with neurotoxicity of cisplatin in TC.	76
BLCA, STAD	Liu et al., 2021	Database analysis, Statistical analysis, etc.	1. The high expression of FAM20C is associated with poor prognosis of BLCA patients and STAD patients. 2. he expression of FAM20C is positively associated with immune infiltration level in STAD and BLCA. 3. The high expression of FAM20C can significantly promote the lymph node metastasis of STAD.	33

CRC, colorectal cancer; BLCA, bladder urothelial carcinoma; STAD, stomach adenocarcinoma; MLM, melanoma; LUAD, lung adenocarcinoma; TC, testicular cancer; OPN, osteopontin; IGF, insulin-like growth factor; IGFBP, insulin-like growth factor-binding protein; GO, Gene Ontology; KEGG, Kyoto Encyclopedia of Genes and Genomes; PPI, protein–protein interaction.

## The role of FAM20C in mineralized tissues

**Figure 4:**
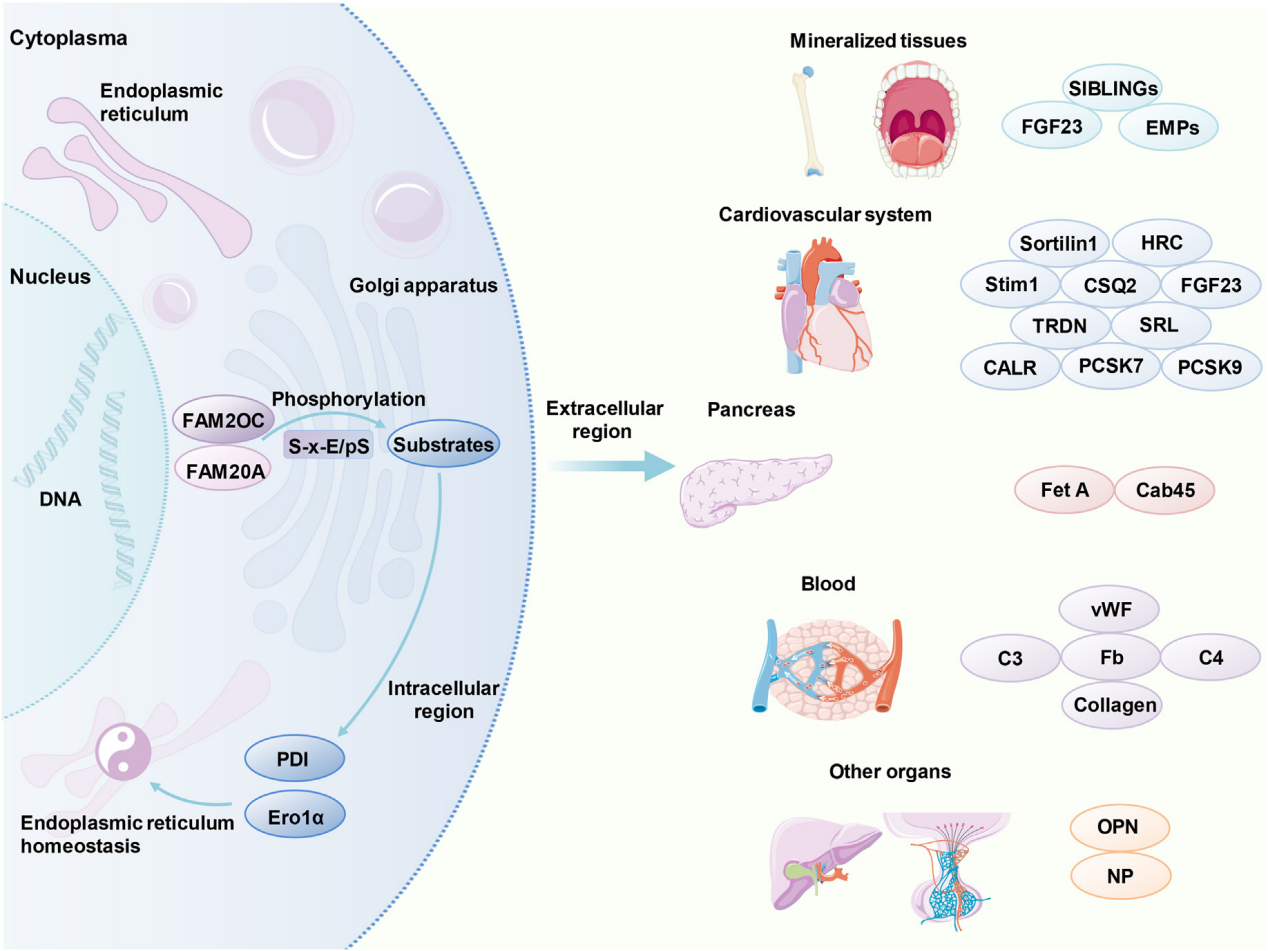
FAM20C and its substrates in noncancerous biological processes. FAM20C plays important roles in several types of organs. FAM20C and related substrates are involved in multiple processes, including biomineralization, myocardial calcium circulation, cardiovascular calcification, lipid and cholesterol regulation, endoplasmic reticulum (ER) homeostasis, blood glucose regulation, coagulation process, liver injury, and neuropeptide secretion.

FAM20C plays significant roles in the mineralization processes of bone and teeth. FAM20C can phosphorylate SIBLING family members, several enamel-matrix proteins, and fibroblast growth factor 23, thus contributing to the mineralization processes (Fig. 4).^6^^,^^7^^,^^81^ SIBLING family includes OPN, bone sialoprotein, dentin matrix protein 1, dentine sialophosphoprotein, and matrix extracellular phosphoglycoprotein.^14^^,^^23^^,^^82^ Enamel-matrix proteins include ameloblastin, amelotin, and enamelin.^83^ These are two groups of extracellular matrix proteins that are essential for bone and tooth formation and play important roles in the process of bone and tooth mineralization. However, the effects of these phosphoproteins on mineralization tissues are multifaceted. For example, OPN and matrix extracellular phosphoglycoprotein inhibit the mineralization process, while dentin matrix protein 1 promotes the process.^84^, ^85^, ^86^, ^87^ Abnormal phosphorylation of these proteins can lead to impaired mineralization. Fibroblast growth factor 23, secreted by osteoblasts, can inhibit phosphate reabsorption and synthesis of 1,25 dihydroxycholecalciferol, thereby regulating phosphate balance and bone development.^88^ In addition, FAM20C can promote the differentiation of osteoblasts, odontoblasts, and ameloblasts.^89^ In conclusion, FAM20C can affect mineralization processes by phosphorylating related proteins, regulating phosphate homeostasis, and promoting cellular differentiation.^89^, ^90^, ^91^

## FAM20C and Raine syndrome

Raine syndrome is an autosomal recessive disorder caused by *FAM20C* gene mutations. It is characterized by generalized osteosclerosis, periosteal bone formation, and ectopic calcification.^13^^,^^92^^,^^93^ Raine syndrome is extremely rare. Most patients die within the first weeks of life because of pulmonary hypoplasia, and a small number of patients survive to adulthood.^94^^,^^95^ Therefore, it includes two types: lethal and non-lethal Raine syndrome. Pathogenic variants of *FAM20C* gene are the cause of Raine syndrome. Many types of variants have been reported, including missense, nonsense, splicing defects, truncated proteins by indels out of frame, and chromosomal rearrangements.^12^^,^^96^, ^97^, ^98^, ^99^

*FAM20C* gene variants that are related to Raine syndrome can occur within or outside the kinase activity domain of FAM20C, so it is assumed that abnormal phosphorylation of SIBLINGs resulting from impaired kinase activity promotes the occurrence and development of Raine syndrome.^14^ In animal experiments, mouse models with OPN or matrix extracellular phosphoglycoprotein knockout both exhibit osteosclerosis.^84^^,^^100^ *In vitro*, OPN reduces crystal development and mineral formation, and it may also help with osteoclast recruitment.^101^ Matrix extracellular phosphoglycoprotein also has a variety of effects *in vitro*, including anti-osteogenic effect and inhibition of phosphate uptake by the intestine and kidney.^102^^,^^103^ However, the specific mechanism of Raine syndrome is not very clear.

Some studies have also reported that environmental factors and oligogenic effects may be implicated in the occurrence and development of Raine syndrome.^104^ It deserves further study. In addition, a major focus of Raine syndrome research in the future is to find the phenotype that corresponds to the genotype to enhance our understanding of the disease.

## FAM20C and hypophosphatemic rickets

Hypophosphatemic rickets are a group of bone mineralization disorders caused by hereditary or acquired factors.^105^ It is characterized by renal phosphate loss and hypophosphatemia.^105^

During mouse osteogenesis, FAM20C mRNA can be observed in chondrocytes and osteoblasts and FAM20C protein in the extracellular matrix.^106^ The global or specific knockout of FAM20C in mineralized tissues in mice can result in hypophosphatemic rickets, along with elevation of fibroblast growth factor 23 and down-regulation of osteoblast differentiation markers.^89^ FAM20C knockout causes elevated fibroblast growth factor 23 levels and then leads to a reduction in the levels of serum phosphate and 1,25 dihydroxycholecalciferol.^12^ *In vitro*, FAM20C overexpression in osteoblasts promotes cortical bone formation and osteoclastic bone resorption in cortical and trabecular bones.^107^ Conversely, FAM20C knockout in osteoblasts reduces the abilities of osteoblasts to migrate and form mineralized nodules, and meanwhile, it causes mesenchymal-to-epithelial transition.^108^^,^^109^

It is suggested that the FAM20C- fibroblast growth factor 23-phosphate pathway and osteoblast differentiation disorder play important roles in the occurrence and development of hypophosphatemic rickets.

## FAM20C and dental diseases

FAM20C is associated with dentin and enamel formation. FAM20C deficiency can cause dentinogenesis imperfecta and amelogenesis imperfecta. During mouse odontogenesis, FAM20C mRNA can be observed in odontoblasts and FAM20C protein in the dentin matrix.^106^ Sox2-Cre; FAM20C^fl/fl^ mice, in which FAM20C is generally inactivated, and Wnt1-Cre; FAM20C^fl/fl^ and Osr2-Cre; FAM20C^fl/fl^ mice, in which FAM20C is inactivated in the craniofacial mesenchymal cells, both have dentin defects.^110^ *In vitro*, FAM20C overexpression can promote odontoblast differentiation and mineralization processes.^89^ FAM20C facilitates odontoblastic differentiation of human dental pulp cells and then accelerates dentin formation.^111^ Moreover, FAM20C in dental mesenchymal cells is crucial for dentin formation.^112^ FAM20C is also related to enamel formation. During odontogenesis, FAM20C mRNA can be observed in ameloblasts and FAM20C protein in the enamel matrix.^106^ Sox2-Cre; FAM20C^fl/fl^ mice also show enamel defects.^110^ K14-Cre; FAM20C^fl/fl^ mice, in which FAM20C is inactivated in the epithelial cells that are in charge of creating tooth enamel have severe enamel and ameloblast defects, but their dentin are not significantly influenced.^83^

In addition, FAM20C can affect periodontal tissues including the periodontal ligament, cementum, alveolar bone, and gingiva. Type I collagen and periostin are the main proteins in the periodontal ligament.^113^^,^^114^ Fibroblasts can produce type I collagen. During odontogenesis, FAM20C mRNA can also be detected in fibroblasts.^106^ FAM20C inactivation in the cells that express type I collagen can cause periodontal defects in 2.3 kb Col 1a1-Cre; FAM20C^fl/fl^ mice, accompanied by inflammation.^113^ Periostin promotes the interaction between cells and extracellular matrix, regulates type I collagen formation, interacts with other extracellular matrix proteins, and plays an important role in the connection and distribution of extracellular matrix.^115^ In periostin knockout mice, fibroblasts are irregularly distributed in the collagen fibers, and the collagen fibers are disorganized.^116^ A recent study has found that periostin is a substrate of FAM20C.^117^ Therefore, it is suggested that FAM20C can affect the periodontal tissues by periostin.

## The role of FAM20C in the cardiovascular system

Several substrates of FAM20C have been identified to play significant roles in the cardiovascular system. Sortilin 1, encoded by the *SORT1* gene, is a type 1 transmembrane glycoprotein receptor that belongs to the vacuolar protein sorting 10 protein domain receptor family.^118^^,^^119^ It is widely expressed in tissues such as the liver, vascular tissue, heart, skeletal muscle, and brain, and has diverse functions.^12^ Notably, sortilin1 acts as a key regulator of vascular calcification and contributes to the formation of calcified atheroma.^120^ As a substrate of FAM20C, it is suggested that FAM20C may be involved in the formation of atherosclerotic calcification lesions through Sortilin1 (Fig. 4).^12^^,^^120^

The sarcoplasmic reticulum (SR) of the myocardium can regulate cardiac contraction and relaxation through the timely release and recycling of Ca^2+^. FAM20C is expressed in the SR and phosphorylates multiple proteins that are involved in Ca^2+^ regulation, including histidine-rich Ca^2+^-binding protein (HRC), calsequestrin 2, stromal interaction molecule 1, sarcalumenin, calreticulin, and triadin (Fig. 4).^121^^,^^122^ HRC participates in the regulation of Ca^2+^ in the SR.^123^ Stress-induced cardiomyocytes after contractions are increased when HRC is depleted.^123^ *HRC* gene mutation is associated with arrhythmia, for example, the Ser96alanine variant in HRC results in an increased risk of ventricular arrhythmias in patients with dilated cardiomyopathy.^124^ The phosphorylation of HRC-Ser96 by FAM20C can help maintain SR Ca^2+^ homeostasis and prevent arrhythmias.^121^^,^^125^ Calsequestrin 2 is the major Ca^2+^ binding protein in the SR, and its phosphorylation by FAM20C can increase the ability of calsequestrin 2 to oligomerize.^122^ Stromal interaction molecule 1 which is a Ca^2+^ sensor in the lumen of the ER/SR in multiple cell types is associated with store-operated Ca^2+^ entry.^122^ FAM20C knockout in cardiomyocytes can promote the occurrence of mouse heart failure caused by aging and stress overload, which is due to impaired Ca^2+^ regulation in the SR.^122^ Furthermore, in patients with chronic renal disease, high levels of fibroblast growth factor 23 are associated with cardiovascular complications and higher mortality.^126^ In conclusion, FAM20C plays crucial roles in the regulation of Ca^2+^ homeostasis and cardiac pathophysiology through phosphorylating related substrates.

Proprotein convertase subtilisin/kexin type 7 (PCSK7) and type 9 (PCSK9) belong to the secretory basic amino acid-specific PCSKs. PCSK7 is expressed in various tissues and is associated with the regulation of triglyceride, which is a known risk factor for heart disease.^12^ FAM20C phosphorylates PCSK7, thus causing decreased degradation of apolipoprotein A-V in the liver, increased lipoprotein lipase activity, increased triglyceride uptake in adipocytes, and ultimately decreased triglyceride levels.^127^ Interestingly, a low-frequency coding variant of *PCSK7* gene, known as rs142953140, can increase its phosphorylation by FAM20C, resulting in lower triglyceride levels.^127^ On the other hand, PCSK9 is highly expressed in the liver and associated with the regulation of cholesterol.^12^ The mutation that affects the S-x-E/pS motif of PCSK9 can lead to the dysregulation of low-density lipoprotein (LDL) cholesterol, which is also a risk factor for heart disease.^128^ LDL cholesterol can be degraded by LDL receptors in the liver. The phosphorylation of PCSK9 by FAM20C increases PCSK9 secretion, enhances the degradation of LDL receptors, and then affects LDL cholesterol level.^128^ It is suggested that FAM20C may regulate the levels of triglyceride and LDL cholesterol through PCSKs, thus influencing the risk factors for heart diseases (Fig. 4).

## The role of FAM20C in ER homeostasis and diabetes

FAM20C participates in the maintenance of ER proteostasis and redox homeostasis, which can prevent potential damage caused by ER stress. Protein disulfide isomerase is one of the most important folding catalysts in the ER.^129^ FAM20C is capable of phosphorylating protein disulfide isomerase-Ser357, thereby facilitating proper protein folding and maintaining appropriate early ER stress responses (Fig. 4).^129^ ER oxidoreductin 1α is an important sulfhydryl oxidase in the ER.^16^ ER oxidoreductin 1α-Ser145 can be phosphorylated by FAM20C in the Golgi apparatus and then retrograde-transported to the ER.^16^ The phosphorylation of ER oxidoreductin 1α increases its oxidase activity, thus regulating the correct folding of oxidative proteins in the secretory pathway and preserving ER redox homeostasis (Fig. 4).^16^

FAM20C phosphorylation plays important roles in the secretion of certain proteins. FAM20C can phosphorylate calcium-binding protein 45 kDa and then regulate its oligomerization and promote the secretion and sorting of its client proteins (Fig. 4).^87^ FAM20C may be a key component in the cellular stress response to changes in blood glucose levels. In type 2 diabetic obese mice, the FAM20C levels in pancreatic β-islet cells are increased under hyperglycemia conditions.^130^ FAM20C can phosphorylate related proteins involved in the secretion and transportation of insulin, thus regulating blood glucose.^130^ Notably, the FAM20C levels return to normal following euglycemia.^130^ Phosphorylated Fet A can inhibit insulin signal transduction by binding to the β-subunit of the insulin receptor, thus causing insulin resistance in type 2 diabetes mellitus.^44^ In addition, Fet A can induce apoptosis signals in pancreatic β-islet cells and then decrease insulin secretion and promote type 2 diabetes mellitus progression.^44^ Fet A is a phosphorylated substrate of FAM20C, indicating that FAM20C may be involved in the development of insulin resistance through Fet A (Fig. 4).^36^

## The role of FAM20C in blood

Phosphoproteomic analyses have revealed that the majority of phosphorylation sites of proteins in plasma and serum are situated in the S-x-E/pS motif, suggesting that these phosphoproteins may be substrates of FAM20C.^131^ Multiple substrates of FAM20C such as fibrinogen, von Willebrand factor, partial complement components, and collagen have been found to be involved in the coagulation process and complement pathways (Fig. 4).^5^^,^^132^^,^^133^ Fibrinogen that is produced by hepatic cells is a key protein in the coagulation process, where it is cleaved by thrombin into fibrin peptides, leading to the formation of a fibrin-based blood clot and the cessation of bleeding. A Study has shown that phosphorylated fibrinogen exhibits better interaction with thrombin, thus being cleaved into fibrin peptide and achieving rapid hemostasis.^134^ von Willebrand factor mediates the adhesion of platelets to vascular injury and facilitates hemostasis. FAM20C can phosphorylate two Ser residues of von Willebrand factor, thereby enhancing platelet adhesion and promoting coagulation.^133^ In addition, complement components 3/4 and collagen are also confirmed to be substrates of FAM20C.^5^ Taken together, these findings indicate that FAM20C participates in the coagulation process through fibrinogen and von Willebrand factor, and may affect other biological processes through complement components and collagen.

## Other roles of FAM20C

Neuropeptides are crucial signaling molecules that play roles in the nervous and endocrine systems. Neuropeptides can be phosphorylated by FAM20C, so FAM20C may be very important for the functions of nervous and endocrine systems (Fig. 4).^135^ The behavioral variant frontotemporal dementia is a clinical syndrome characterized by progressive declines in personality, social behavior, and cognitive function caused by neurodegeneration of the frontal and/or temporal lobes. Compared with patients with such dementia but no autoimmune disease, serum FAM20C levels are reduced in patients with such dementia and autoimmune disease.^136^ Additionally, FAM20C promotes OPN secretion from hepatic stellate cells during liver injury. OPN can be secreted into the extracellular matrix to enhance the formation of liver fibrosis (Fig. 4).^137^ Furthermore, FAM20C inactivation in the salivary gland cells of FAM20C^f/f^;Mmtv-Cre mice can result in a decrease in the number of acinar cells and structural abnormalities in the ducts of the salivary gland.^138^

## Conclusions and future perspectives

FAM20C, as a protein kinase, has been identified to play crucial roles in cancer progression, mineralized tissue formation, cardiovascular pathophysiological changes, and other processes. The unclear specific mechanisms necessitate further research to comprehend the roles of FAM20C in related diseases. Consequently, the development of drugs that target FAM20C could bring new hope for the prevention and treatment of related diseases. Especially in malignant tumors, a large number of researchers have confirmed that FAM20C participates in the invasion and metastasis of glioma, BRC, and other cancers. Thus, we strongly believe that FAM20C-targeting drugs possess great potential to inhibit cancer progression and improve the quality of life and prognosis of patients.

## Conflict of interests

The authors declare no conflict of interests.

## Funding

Y.W. is supported by the National Key Research and Development Program of China, Stem Cell and Translational Research (2022YFA1105200), the National Natural Science Foundation of China (82273117), and Sichuan Science and Technology Program (23ZYZYTS0150). Y.Z. is supported by the National Natural Science Foundation of China (82173179) and the National Clinical Research Center for Geriatrics (Z2021JC006). YM.R. is supported by the National Natural Science Foundation of China (82302627) and the Science and Technology Supportive Project of Sichuan Province (2022YFS0143). YK.Z. is supported by the Science and Technology Supportive Project of Sichuan Province (2022YFS0049).
